# Integrated Analysis of Transcriptome Profiles and lncRNA–miRNA–mRNA Competing Endogenous RNA Regulatory Network to Identify Biological Functional Effects of Genes and Pathways Associated with Johne’s Disease in Dairy Cattle

**DOI:** 10.3390/ncrna10040038

**Published:** 2024-06-28

**Authors:** Farzad Ghafouri, Vahid Dehghanian Reyhan, Mostafa Sadeghi, Seyed Reza Miraei-Ashtiani, John P. Kastelic, Herman W. Barkema, Masoud Shirali

**Affiliations:** 1 Department of Animal Science, College of Agriculture and Natural Resources, University of Tehran, Karaj 77871-31587, Iran; farzad.ghafouri@ut.ac.ir (F.G.); vahid.dehghaniya@ut.ac.ir (V.D.R.); ashtiani@ut.ac.ir (S.R.M.-A.); 2 Faculty of Veterinary Medicine, University of Calgary, Calgary, AB T2N 4N1, Canada; jpkastel@ucalgary.ca (J.P.K.); barkema@ucalgary.ca (H.W.B.); 3 School of Biological Sciences, Queen’s University Belfast, Belfast BT9 5AJ, UK; 4 Agri-Food and Biosciences Institute, Hillsborough BT26 6DR, UK

**Keywords:** paratuberculosis, MAP infection, ceRNA regulatory network, transcriptome analysis, immune responses, dairy cattle

## Abstract

Paratuberculosis or Johne’s disease (JD), a chronic granulomatous gastroenteritis caused by *Mycobacterium avium* subsp. *paratuberculosis* (MAP), causes huge economic losses and reduces animal welfare in dairy cattle herds worldwide. At present, molecular mechanisms and biological functions involved in immune responses to MAP infection of dairy cattle are not clearly understood. Our purpose was to integrate transcriptomic profiles and competing endogenous RNA (ceRNA) network analyses to identify key messenger RNAs (mRNAs) and regulatory RNAs involved in molecular regulation of peripheral blood mononuclear cells (PBMCs) for MAP infection in dairy cattle. In total, 28 lncRNAs, 42 miRNAs, and 370 mRNAs were identified by integrating gene ontology (GO) and Kyoto Encyclopedia of Genes and Genomes (KEGG) enrichment analyses. In this regard, we identified 21 hub genes (*CCL20*, *CCL5*, *CD40*, *CSF2*, *CXCL8*, *EIF2AK2*, *FOS*, *IL10*, *IL17A*, *IL1A*, *IL1B*, *IRF1*, *MX2*, *NFKB1*, *NFKBIA*, *PTGS2*, *SOCS3*, *TLR4*, *TNF*, *TNFAIP3*, and *VCAM1*) involved in MAP infection. Furthermore, eight candidate subnets with eight lncRNAs, 29 miRNAs, and 237 mRNAs were detected through clustering analyses, whereas GO enrichment analysis of identified RNAs revealed 510, 22, and 11 significantly enriched GO terms related to MAP infection in biological process, molecular function, and cellular component categories, respectively. The main metabolic-signaling pathways related to MAP infection that were enriched included the immune system process, defense response, response to cytokine, leukocyte migration, regulation of T cell activation, defense response to bacterium, NOD-like receptor, B cell receptor, TNF, NF-kappa B, IL-17, and T cell receptor signaling pathways. Contributions of transcriptome profiles from MAP-positive and MAP-negative sample groups plus a ceRNA regulatory network underlying phenotypic differences in the intensity of pathogenicity of JD provided novel insights into molecular mechanisms associated with immune system responses to MAP infection in dairy cattle.

## 1. Introduction

Paratuberculosis or Johne’s disease (JD) is one of most economically important diseases of the dairy industry globally [1,2]. The causative agent, *Mycobacterium avium* subsp. *paratuberculosis* (MAP), induces chronic granulomatous gastroenteritis in cattle [3]. Infected cattle have chronic diarrhea, weakness, weight loss, a gradual decline in milk production, decreased fertility, and eventual death [2,4,5]. Economic losses and challenges caused by this disease in the dairy industry have been increasing. In a survey of 48 countries, nearly half had >20% of herds infected with the disease [1,5]. In European countries, 31 to 71% of dairy cattle herds are infected [6], and in the United States, JD was detected in > 68% of dairy herds [7]. The disease inflicts substantial economic losses on the dairy industry annually, with estimated losses of USD 200 million to USD 1.5 billion in the US [8] and >USD 35–57 per cow in Canada [9]. Furthermore, the causal role of MAP in the pathogenesis of Crohn’s disease, a chronic inflammatory disease of the human intestine, is controversially discussed [3,10].

Macrophages and dendritic cells (DCs) constitute peripheral blood mononuclear cells (PBMCs) derived from monocytes that move into sites of infection in response to inflammatory signals [11]. The interaction between MAP and macrophages is critical in determining whether the host can clear the pathogen, or if infection will be established. Control of MAP infection depends on several factors, including an early protective Th1 cell response that stimulates IFN-γ release and activates macrophages’ antimicrobial mechanisms [4,12,13,14].

As JD is incurable, early detection and isolation of infected animals prior to shedding is critical to reduce disease incidence. Fecal PCR and serum ELISA have been widely used to detect infected cattle, but their sensitivity is low, as shedding occurs intermittently, and serum antibody titers against MAP only increase in late stages of infection [3,4,15,16,17]. Economic losses, animal welfare concerns, and potential zoonotic risks associated with JD have driven research to improve early diagnosis and new diagnostic techniques [18,19]. Characterizing expression profiles of RNA molecules (coding and noncoding) in host macrophages during infection could clarify molecular mechanisms and host-pathogen interactions associated with JD. The mRNA expression profile of bovine macrophage responses during inflammation from MAP infection has been described [20,21]. Genetic variations in several candidate genes expressed in macrophages were associated with resistance/susceptibility to MAP infection, including NOD2 [22], IL10 [23,24,25], SLC11A1, and Toll-like receptor genes [26,27].

The use of non-coding RNAs (ncRNAs) as a novel and promising diagnostic approach for infectious and non-infectious diseases, is of increasing interest. Bacteria can interfere with expression of mammalian regulatory RNAs to modulate immune signaling, autophagy, and apoptotic pathways [6,28,29,30,31]. Long non-coding RNAs (LncRNAs) are a class of ncRNAs with >200 nucleotides that are predominantly transcribed from the antisense strand and intergenic spacer regions of protein-coding genes [32]. LncRNAs are involved in various diseases and are being explored as potential molecular biomarkers for disease diagnosis and treatment, especially in host cell responses to bacterial infections [33,34,35]. MicroRNAs (miRNAs) are short, ncRNAs, typically 19–24 nucleotides in length, that bind to the 3′ untranslated regions of target mRNAs to regulate their translation into proteins or promote mRNA decay. Many studies have reported the role of miRNAs in a wide range of cellular processes, including cell proliferation, differentiation, and apoptosis [10,36]. Additionally, some research indicates that miRNAs regulate both innate and adaptive immune mechanisms [37]. In high-throughput sequencing of small RNA libraries of CPIV3-infected and mock-infected bovine kidney epithelial (MDBK) cells, 249 known and 152 novel differential expression (DE) miRNAs were identified [38]. Both miRNAs and lncRNAs can function as competitive endogenous RNAs (ceRNAs) to influence gene expression by modulating pathophysiological conditions in various organisms [39]. Analyses of integrated lncRNA-miRNA-mRNA ceRNA networks have provided insights into complex molecular mechanisms by accounting for crosstalk between various regulatory RNAs [40,41,42].

This study consolidated information on a diverse range of RNA molecules and their role as ceRNAs, using transcriptome profiles and literature mining to address research questions regarding biological and regulatory functional effects of genes, ncRNAs, and pathways associated with JD in dairy cattle. A better understanding of the pathogenic mechanisms of MAP infection is very important for early diagnosis, vaccine development, and improved control of JD progression. Multi-part network-based approaches integrating various components of the transcriptome have offered new perspectives on regulation of complex multigenic traits [41,42]. However, few studies have focused on non-coding RNAs associated with JD. Therefore, it was considered essential to conduct a comprehensive study, integrating transcriptional profiles, literature mining (to encompass findings of previous studies), ceRNA regulatory networks, and their subnets. Comparative transcriptomic analyses of bovine PBMCs of MAP-infected and control cattle were conducted to identify related mRNAs/genes, lncRNAs, and miRNAs, their functions, and important pathways. In addition, a lncRNA–miRNA–mRNA ceRNA regulatory network was constructed and its subnets were determined to better understand molecular mechanisms involved in JD of dairy cattle.

## 2. Results

### 2.1. Identified DE RNAs from RNA-Seq and Microarray Data Analysis

**Figure 1:**
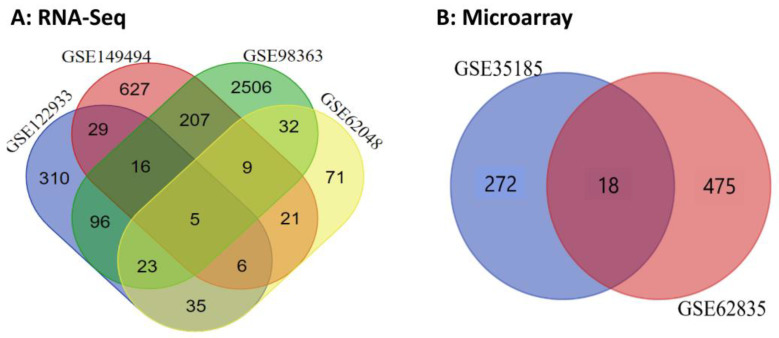
Venn diagram of common significant genes for (**A**) RNA-Seq and (**B**) microarray datasets related to MAP infection in dairy cattle.

To reconstruct the lncRNA–miRNA–mRNA ceRNA regulatory network and better understand the molecular mechanisms and genetic basis of JD, transcriptome profiles of PBMCs from MAP-positive versus MAP-negative samples were compared. In this study, the experimental data, RNA-Seq and microarray datasets, were selected from the GEO database (see Section 4). The mRNAs/genes differentially expressed between MAP-positive and MAP-negative samples were identified with a threshold of |log2 fold change| ≥ 1 and FDR ≤ 0.05. In statistical analyses of microarray datasets, 783 significant DE mRNAs/genes were obtained from two datasets, with 290 and 493 DE mRNAs/genes in the first (GSE35185) and second (GSE62835) datasets, respectively. Also, in the RNA-Seq data analysis, a total of 4536 DE mRNAs/genes were identified, of which 520, 920, 2894, and 202 mRNAs/genes were detected for accession numbers GSE122933, GSE149494, GSE98363, and GSE62048, respectively (Supplementary Table S1). Finally, in the Venn analysis, eighteen and five DE mRNAs/genes were identified as common DE mRNAs/genes for microarray and RNA-Seq datasets, respectively (Supplementary Table S3 and Figure 1). In this regard, 18 genes were identified as common genes between GSE35185 and GSE62835 accessions in microarray data analysis, and the *ACSL6*, *CDC42EP4*, *GCH1*, *GJB2*, *IL10*, *PLBD1*, *PTGS2*, *SERPINE1*, *SLC2A6*, *SLC46A2*, *TNFSF13B*, *TTYH2*, and *UPB1* genes were up-regulated in both GSE datasets. *TNFAIP6*, *CCL4*, *NOS2*, *TFPI2*, and *CCL20* genes were mostly up-regulated among all GSE accessions in RNA-Seq data analysis. Furthermore, 19 miRNAs were identified in the RNA-Seq data analysis by considering the threshold of |log2 fold change| ≥ 1 and FDR ≤ 0.05 (Table 1).

### 2.2. Literature Mining and Identification of Main Gene List

A list of candidate ncRNAs potentially involved in JD was created by literature mining. Specifically, 28 candidate lncRNAs and 28 miRNAs were identified by reviewing published literature (Supplementary Table S3). Finally, the main RNAs list was obtained by integrating the DE RNAs list from transcriptome profiling analysis and the ncRNAs list from literature mining (Supplementary Table S4).

### 2.3. Functional Categorization and Pathway Enrichment Analysis of DE mRNAs

**Figure 2:**
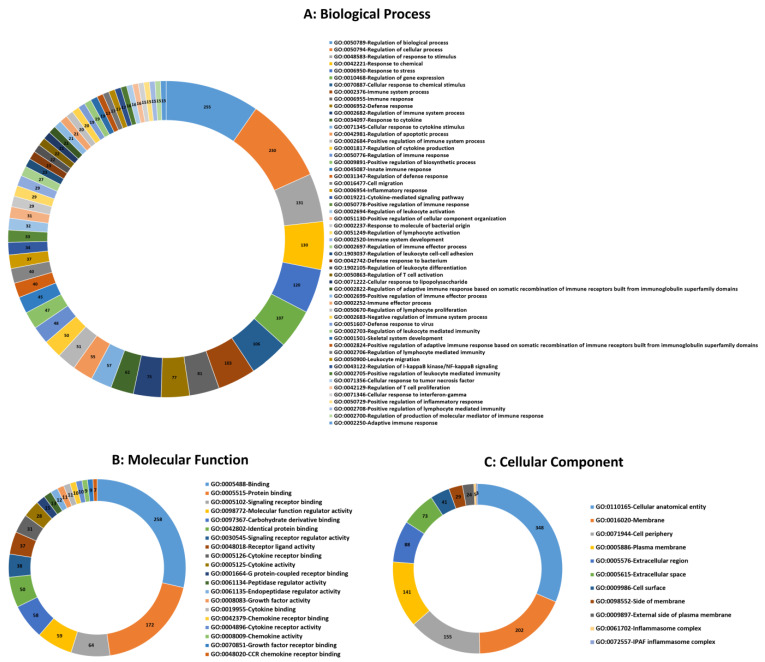
Top significant mRNA/gene ontology (GO) terms including biological process (**A**), molecular function (**B**), and cellular component (**C**) enriched using mRNAs/genes associated with MAP infection in dairy cattle. The numbers indicate the number of genes associated with each pathway.

**Figure 3:**
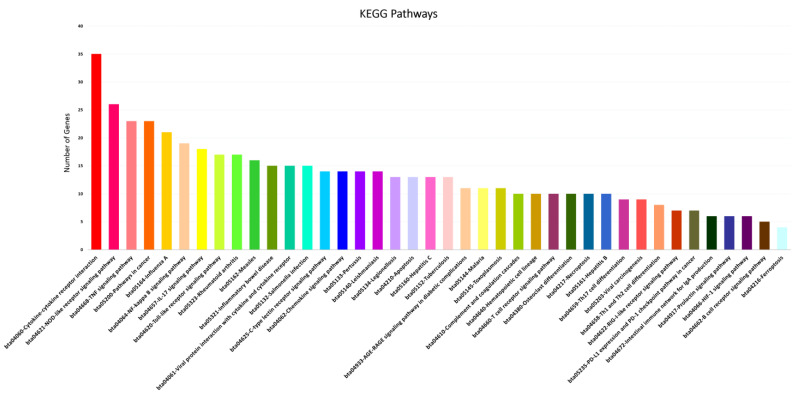
Top KEGG pathways enriched using significant mRNAs/genes associated with MAP infection in dairy cattle.

Functional categorization of GO analysis was performed based on biological process (BP), molecular function (MF), and cellular component (CC) to explore related biological functions of DE mRNAs associated with JD and its pathogenesis (Figure 2, Supplementary Table S6). There were 510 BPs identified; regulation of immune system process, regulation of immune response, defense response, response to cytokine, cellular response to cytokine stimulus, innate immune response, regulation of defense response, leukocyte migration, regulation of I-kappaB kinase/NF-kappaB signaling, regulation of T cell activation, defense response to bacterium, and regulation of leukocyte differentiation were the 12 significant categories associated with MAP infection (Figure 2A). Furthermore, the identified mRNA/genes were enriched in 22 significant MFs, namely protein binding, signaling receptor binding, CCR chemokine receptor binding, cytokine activity, G protein-coupled receptor binding, growth factor activity, cytokine binding, chemokine receptor binding, cytokine receptor activity, and chemokine activity functions identified as top functions involved in immune responses to MAP infection (Figure 2B). For CCs, 11 terms, including cellular anatomical entity, membrane, cell periphery, plasma membrane, extracellular region, extracellular space, cell surface, side of membrane, external side of plasma membrane, inflammasome complex, and IPAF inflammasome complex were identified (Figure 2C). Moreover, based on KEGG pathway analysis of the identified DE mRNAs/genes, 71 signaling pathways were identified, of which the NOD-like receptor, B cell receptor, TNF, NF-kappa B, IL-17, Toll-like receptor, chemokine, T cell receptor signaling pathways, cytokine-cytokine receptor interaction, tuberculosis, Th17 cell differentiation, Th1 and Th2 cell differentiation, and intestinal immune network for IgA production were the most significant pathways associated with MAP infection (Figure 3, Supplementary Table S7).

### 2.4. PPI Network Construction and Hub Genes Determining

The protein-protein interactions (PPI) network, constructed with the STRING database, indicates interactions between proteins (mRNAs) based on biochemical functions. The PPI network generated contained 370 nodes (mRNAs) and 1804 edges representing interactions between nodes (Supplementary Table S1). Moreover, hub-hub mRNAs/genes were identified based on more interactions in the PPI network, including *CCL20*, *CCL5*, *CD40*, *CSF2*, *CXCL8*, *EIF2AK2*, *FOS*, *IL10*, *IL17A*, *IL1A*, *IL1B*, *IRF1*, *MX2*, *NFKB1*, *NFKBIA*, *PTGS2*, *SOCS3*, *TLR4*, *TNF*, *TNFAIP3*, and *VCAM1*.

### 2.5. Reconstruction of lncRNA–miRNA–mRNA ceRNA Regulatory Network

**Figure 4:**
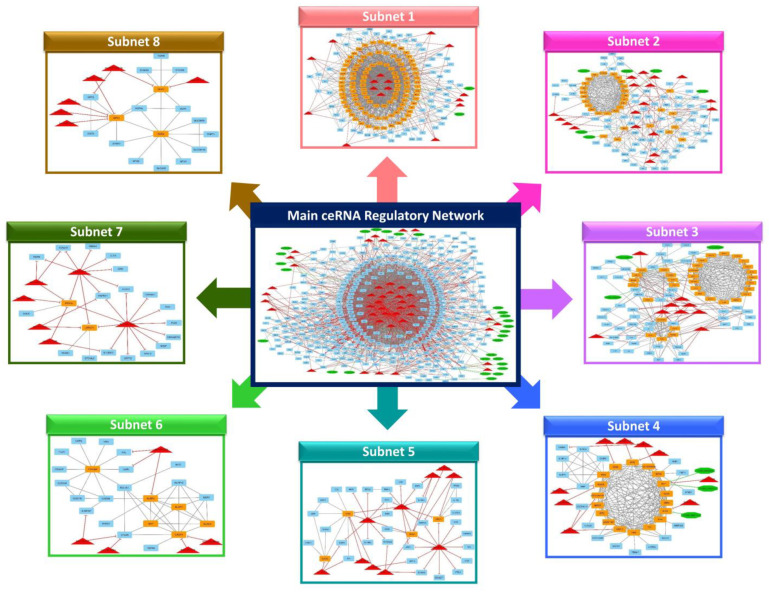
The main lncRNA–miRNA–mRNA ceRNA regulatory network and its subnets. Octagonal nodes represent lncRNAs, triangle nodes represent miRNAs, and quadrilateral nodes represent mRNAs/genes. Edges indicate interactions between nodes.

To understand how ncRNAs (e.g., lncRNAs) regulate gene expression by targeting miRNAs and messenger mRNAs, using knowledge extracted from the STRING online database and other relevant papers via literature mining, a lncRNA–miRNA–mRNA ceRNA regulatory network was reconstructed, integrating regulatory interactions between RNA molecules. This regulatory network involved 440 nodes and 2372 edges. The 440 nodes included 28 lncRNAs, 42 DE miRNAs, and 370 DE mRNAs that were incorporated into the regulatory network (Figure 4, Supplementary Figure S1). Furthermore, bta-mir-125b-1, bta-mir-140, bta-mir-2384, bta-mir-2398, bta-mir-302a, bta-mir-302c, bta-mir-339a, bta-mir-4657, bta-mir-147, bta-mir-371, bta-miR-132, bta-miR-150, bta-miR-214, bta-miR-677, bta-miR-1306, bta-miR-1343-3p, bta-miR-2443, bta-miR-12023, and bta-miR-2887 miRNAs were considered as hub miRNAs, based on their connections in the ceRNA regulatory network.

### 2.6. Clustering Analysis of the lncRNA–miRNA–mRNA ceRNA Regulatory Network

After reconstructing the lncRNA-miRNA-mRNA ceRNA regulatory network, the MCODE plugin of Cytoscape (v 3.8.2) was used for clustering analysis [43]. In total, 8 subnets were generated, including 8 lncRNA, 29 miRNA, and 237 mRNA, considering repeated nodes. In addition, subnets 1 to 8 had 188, 125, 94, 47, 45, 28, 26, and 23 nodes, and 1541, 415, 374, 163, 63, 50, 32, and 31 edges, respectively (Figure 4, Supplementary Figures S1–S9).

Among the subnets, subnet 1 included all hub-hub genes; therefore, it was considered the most important subnet. In subnets 1, 2, and 3, bta-miR-150 and bta-miR-12023 miRNAs suppressed the most genes, whereas bta-miR-1343-3p and bta-miR-2443 were hub miRNAs in subnets 1 and 2. *TNFAIP3* was a hub-hub gene in subnets 1 and 3 and was suppressed by the bta-mir-147 miRNA and TCONS_00066475 lncRNA. Also, the *FSO* gene was suppressed by bta-miR-12023 miRNA in subnets 2 and 3, and the *MX2* gene was suppressed by bta-miR-1306 miRNA as well as TCONS_00002516 and TCONS_00002515 lncRNAs in subnets 2 and 4, and both were hub-hub genes. Moreover, *CSF2*, *CXCL8*, *IRF1*, and *TNF* were among the hub-hub genes presented in subnet 1, and the *CSF2* and *TNF* genes were suppressed by bta-miR-2443 and bta-miR-454 miRNAs, respectively. The *IRF1* gene was suppressed by bta-miR-132, bta-miR-150, and bta-miR-1343-3p miRNAs, and the ENSBTAG00000051518 lncRNA suppressed the *CXCL8* gene. All these genes, except the *FSO* gene, were up-regulated in MAP-infected versus MAP-negative samples.

In subnet 2, jointly with subsets 1, 3, and 4, six genes were presented as hub genes, including *THBD*, *GJA1*, *BIRC3*, *IFIT5*, *IFI44L*, and *RSAD2*. The *THBD* and *GJA1* genes were suppressed by most miRNAs, with bta-miR-150, bta-miR-1434-5p, bta-miR-2443, bta-miR-451, and bta-miR-1343-3p miRNAs suppressing the *THBD* gene, and bta-miR-454, bta-miR-214, bta-miR-1306, and bta-miR-1434-5p miRNAs suppressing the *GJA1* gene. Also, the *BIRC3* gene was a hub gene in subnets 2 and 3. This gene was suppressed by bta-miR-655 and bta-miR-369-3p miRNAs in subnet 3, as well as the TCONS_00016296 lncRNA in subnet 2. Furthermore, the *IFIT5*, *RSAD2*, and *IFI44L* genes were suppressed by bta-miR-1246, bta-miR-150, bta-miR-454, bta-miR-1434-5p, and bta-miR-2315 miRNAs and TCONS_00051144 lncRNA in subnets 2 and 4.

In subnets 5 and 7, bta-miR-677 and bta-miR-2443 were presented as hub miRNAs, whereas bta-miR-2443 was also a hub mRNA in subnet 1 and bta-miR-371 was a hub miRNA in subnet 5. Furthermore, *LRRC71* and *PEG10* genes were among the hub genes suppressed by many miRNAs in subnets 5 and 7. The *PEG10* gene was up-regulated in MAP-positive versus MAP-negative samples, whereas *LRRC71* was down-regulated.

In subnet 6, bta-miR-302a miRNA had a high connection rate compared to other miRNAs and suppressed *AXL*, *LAIR1*, and *NLRP3* genes (that were up-regulated in MAP-positive versus MAP-negative samples), in which the *NLRP3* gene was a hub gene in subnets 1 and 6. Also, the *CASP4* gene was a hub gene in subnets 1 and 6 and was suppressed by bta-miR-380-3p and bta-miR-2443 miRNAs.

In subnet 8, the *GPX3* (glutathione peroxidase 3) gene was a hub gene that was suppressed by most miRNAs, including bta-miR-12023, bta-miR-2315, bta-miR-122, and bta-miR-2384. This gene was up-regulated in MAP-positive versus MAP-negative samples.

## 3. Discussion

Johne’s disease is a chronic and complex disease that has a great deleterious impact on dairy farm profitability [44]. Detection of MAP infection is crucial for JD control; however, current diagnostic tests have poor sensitivity for identifying subclinically infected cattle. Evaluating host biomarkers could lead to improved diagnostic assays for MAP infection and enhance JD control programs [45].

In recent years, high-throughput transcriptomic technologies, plus continuous improvements of genomic resources, have greatly increased the ability to understand interactions between host macrophages and mycobacterial pathogens [5,10,46]. Currently, most transcriptome studies have primarily focused on mRNA analyses; however, miRNAs can regulate mRNA expression during inflammatory responses [10,44,46]. Interactions between various non-coding RNA species such as miRNAs and lncRNAs can provide important insights into molecular mechanisms involved in JD pathogenesis [2,47]. Following presentation of the ceRNA hypothesis [48], many studies were conducted to explain the function of ceRNA (especially lncRNA). However, few studies have addressed interactions of coding and non-coding RNAs through an integrated regulatory network.

According to the ceRNA hypothesis, complementary RNA molecules, such as lncRNAs and circRNAs, can regulate expression of target mRNA/gene, either as a specific miRNA by sponging and targeting it or as a competing endogenous RNA (ceRNA). Attempts have been made to construct ceRNA networks in animal science [41,42,49,50]. In this study, integrative analysis of multiple datasets (RNA-Seq and microarray) and literature mining identified 28 lncRNAs, 47 DE miRNAs, and 5319 DE mRNAs. Then, we recognized potential lncRNA–miRNA–mRNA ceRNA interactions involved in JD in dairy cattle. Ultimately, we reconstructed a lncRNA–miRNA–mRNA ceRNA regulatory network and eight candidate subnets. Key mRNAs/genes were identified as “hub-hubs,” based on having a high degree of connectivity and frequency of appearance between the main ceRNA regulatory network and subnets. Additionally, GO analysis categorized the functional annotation of the DE RNAs into three groups: biological process (510 terms), molecular function (11 terms), and cellular component (22 terms). In addition, in the KEGG pathway analysis, 71 pathways were identified. The majority of the identified metabolic and signaling pathways were associated with inflammatory responses, immune system, and white blood cell activity.

According to GO analysis, most of the common genes between GSEs accessions in microarray and RNA-Seq data analysis separately were involved in three groups (BP, MF, and CC) including regulation of biological processes, cellular response to chemical stimulus, cellular response to cytokine stimulus, regulation of immune system process, innate immune response, cellular response to interferon-gamma, cell migration, regulation of defense response, cytokine-mediated signaling pathway, regulation of leukocyte activation, response to molecule of bacterial origin, protein binding, signaling receptor binding, cytokine receptor binding, G protein-coupled receptor binding, chemokine activity and CCR chemokine receptor binding, cellular anatomical entity, membrane, cell periphery, and extracellular space. Furthermore, these genes were mainly enriched in pathways of cytokine-cytokine receptor interaction, NF-kappa B signaling pathway, and viral protein interaction with cytokine and cytokine receptors. Also, *PTGS2*, *IL10*, *CCL4*, and *CCL20* were present in most metabolic pathways associated with MAP infection. Moreover, *PTGS2*, *IL10*, and *CCL20* genes were among the hub-hub genes in the ceRNA regulatory network. The *PTGS2* (prostaglandin-endoperoxide synthase 2) gene encodes one isozyme of the prostaglandin G/H synthase family. The *PTGS2* gene is responsible for production of inflammatory prostaglandins and is specifically regulated during physiological stress, such as inflammation and infection [51,52]. The protein encoded by the *IL10* (Interleukin 10) gene is a cytokine, primarily produced by monocytes and to a lesser extent lymphocytes. This protein is involved in a variety of immunoregulatory and inflammatory functions, including decreased expression of Th1 cytokines, MHC class II antigens, and other proteins stimulating immune cells such as macrophages, promoting B cell endurance, proliferation, and antibody production and regulating the JAK-STAT signaling pathway. The protein encoded by *CCL20* gene, in binding with *CCR6*, is involved in chemotaxis of dendritic cells (DCs), effector/memory T cells, and B cells, as well as neutrophils, with important roles in skin and mucosal surfaces under homeostatic and inflammatory conditions, as well as in pathology, including cancer and various autoimmune diseases [53,54]. The *CCL4* gene regulates the immune response to infection and inflammation. During inflammation, *CCL4* can induce expression of other pro-inflammatory cytokines, e.g., TNF-α, IL-1β, and IL-6 from activated macrophages and fibroblasts. As a chemoattractant, *CCL4* attracts key regulatory immune cells, including monocytes, macrophages, T cells, natural killer cells, and dendritic cells to sites of inflammation [55,56].

Among subnets, in subnet 1, *TNFAIP3* (Tumor necrosis factor alpha-induced protein 3) gene is a key regulator of NF-kappa B activation and TNF-mediated cell death (apoptosis). The *TNFAIP3* gene controls the levels of proteins inside cells by promoting their degradation via the ubiquitin system. In general, *TNFAIP3* gene expression is temporarily increased in many cell types in response to inflammatory signals and activation of the immune system, implicating *TNFAIP3* in inflammatory and immune responses across cell and tissue types [57,58]. The *FOS* gene family encodes leucine zipper proteins that can dimerize with JUN family proteins to form the AP-1 transcription factor complex. Thus, *FOS* proteins are involved in regulation of the cell cycle, apoptosis, proliferation, and differentiation [59]. The *MX2* gene encodes a protein that belongs to the dynamin and GTPase families. The *MX2* protein has a critical role in antiviral responses; it is induced by interferon-alpha (INF-α) and exhibits potent activity against HIV-1 infection [60]. Studies by Kane et al. [61] further demonstrated that decreasing *MX2* levels substantially reduce anti-HIV effects of interferon-alpha. This confirms that *MX2* is a key effector molecule mediating antiviral effects of interferon-alpha against HIV-1. *CSF2* (Colony-stimulating factor 2) gene, also known as granulocyte-macrophage colony-stimulating factor (GM-CSF), is a cytokine that controls production, differentiation, and function of granulocytes and macrophages. This gene has a role in promoting tissue inflammation. GM-CSF signaling impacts survival and activation of myeloid cells, dendritic cell differentiation, and polarization towards the M1 macrophage phenotype. In addition, it also boosts antigen presentation, induces phagocytosis, recruits monocytes and other myeloid cell types from the bone marrow into circulation, and promotes chemotaxis [62,63,64]. The protein encoded by the *IRF1* gene is a transcriptional regulator and tumor suppressor. It activates the transcription of genes involved in both innate and adaptive immune responses, with roles in cell proliferation, apoptosis, immune responses, and DNA damage responses. Specifically, this protein activates genes involved in antiviral and antibacterial defenses [65,66,67]. *TNF* (Tumor necrosis factor; also called TNFα) gene is a multifunctional cytokine regulating various cellular processes, including cell survival, proliferation, differentiation, and apoptosis. This proinflammatory cytokine is produced by immune cells and can be involved in inflammation-related carcinogenesis [68,69]. *CXCL8* (interleukin-8 or IL-8) gene is the most potent neutrophil-attracting chemokine and has a major role as a mediator of the inflammatory response. This protein is secreted by most immune cells, epithelial cells, and fibroblasts and acts as a chemotactic agent by directing neutrophils to the site of infection. Bacterial and viral products rapidly induce *CXCL8* gene expression [70,71]. These genes were mainly involved in three groups (BP, MF, and CC), including regulation of biological process, regulation of cellular process, regulation of gene expression, cellular response to chemical stimulus, immune system process, immune response, response to cytokine, cellular response to cytokine stimulus, regulation of cytokine production, cytokine-mediated signaling pathway, immune system development, protein binding, and cellular anatomical entity. Furthermore, pathways mostly affected by these genes included cytokine-cytokine receptor interaction, influenza A, Pertussis, NOD-like receptor, TNF, NF-kappa B, and IL-17 signaling pathways. Among these signaling pathways, NOD (nucleotide-binding oligomerization domain)-like receptors are important components in the innate immune system of mammals, regulating the immune response and inflammatory response [72,73]. The TNF signaling pathway has a critical role in modulating immune cell responses as it can elicit a multitude of effects, including fever, apoptosis, cachexia, inflammation, suppressed tumor growth and viral replication, and sepsis responses through induction of IL1 and IL6 [74,75]. The NF-κB signaling pathway regulates many critical cellular processes, including infection, inflammation, immune response, apoptosis, tumor, cell cycle regulation, and cell differentiation through target gene expression products. This pathway is conventionally activated by TNF-α, IL-1, or byproducts of bacterial and viral infections [76,77]. The IL-17 signaling pathway is involved in host defense against extracellular bacterial and fungal infections and various autoimmune diseases [78,79].

In addition, we discovered the *IL1A*, *IL1B*, *IL17A*, *NFKBIA*, and *NFKB1* hub-hub genes were present in most terms and pathways associated with MAP infection. These genes were up-regulated in MAP-positive compared to MAP-negative samples. The interleukin-1 alpha (*IL1A*) protein, a multifunctional cytokine belonging to the interleukin-1 family, is produced (by monocytes and macrophages) as an inactive precursor that becomes activated in response to cell damage. After processing and secretion, by inducing cell apoptosis, *IL1A* helps to eliminate damaged cells and resolve inflammation [80,81]. The pro-inflammatory cytokine interleukin-1 beta (IL1B) has a central role in host immune defenses and wound healing. Macrophages activated during infection or injury produce *IL1B* as an inactive precursor. *IL1B* signaling orchestrates key aspects of innate immunity and the inflammatory response to pathogens and tissue damage [82,83,84]. The *IL17A* gene is a member of the IL-17 receptor family and a proinflammatory cytokine produced by activated T cells. This cytokine induces production of inflammatory molecules, chemokines, antimicrobial peptides, and remodeling proteins, with important roles in host defense, cell trafficking, immune modulation, and tissue repair, plus a key role in induction of innate immune defenses [85,86]. The *NFKBIA* gene encodes a subunit of the IKK protein complex. This protein complex binds to DNA and regulates the activity of multiple genes, including those that control the body’s immune responses and inflammatory reactions. Furthermore, it protects the cell from specific signals that would otherwise trigger the cell to self-destruct through programmed cell death (apoptosis) [87,88]. The *NFKB1* gene encodes a constituent subunit of the NF-kappa-B (NFKB) protein complex. NF-kappa-B is a pleiotropic transcription factor that translocates into the nucleus and stimulates expression of genes involved in a wide variety of biological functions, including inflammation, immunity, differentiation, cell growth, tumorigenesis, and apoptosis [89].

In addition, we identified the *SERPINE1* gene as a hub gene in subnets 1 and 3 suppressed by most miRNAs including bta-miR-212, bta-miR-150, bta-miR-1343-3p, and bta-miR-2443 miRNAs, and up-regulated in MAP-positive versus MAP-negative samples. The *SERPINE1* gene contains instructions to produce plasminogen activator inhibitor 1 protein (PAI-1), which has an important role in blood clotting. When an injury occurs, blood clots plug damaged blood vessels and mitigate blood loss. Furthermore, PAI-1 may be involved in cell migration and tissue remodeling [90,91]. This gene was involved in regulating biological process, macromolecule metabolic process, multicellular organismal process, protein metabolic process, responses to external stimulus, angiogenesis, protein binding, molecular function regulator activity, cellular anatomical entity, and extracellular space [92,93].

In subnet 2, jointly with subsets 1, 3, and 4, *THBD* (thrombomodulin) gene is an anticoagulant transmembrane factor expressed in endothelial cells that forms a 1:1 stoichiometric complex with thrombin, leading to transformation of protein C into its activated form. *THBD* binds thrombin and promotes cleavage of protein C and thrombin activatable fibrinolysis inhibitor (TAFI), thereby inhibiting coagulation and fibrinolysis [94,95]. The *GJA1* gene contains instructions to make connexin 43, a connexin family protein with a role in cell-to-cell communication by forming channels between cells, enabling transport of nutrients, ions, and other small signaling molecules [96,97]. The *BIRC3* gene encodes a member of the inhibitor of apoptosis (IAP) family of proteins that inhibit cell death (apoptosis) by binding to tumor necrosis factor receptor-associated factors TRAF1 and TRAF2 [98,99]. The protein encoded by the *IFIT5* gene is an important antiviral factor that recognizes viral RNA patterns, triggers innate immune defenses, and employs multiple mechanisms to inhibit viral replication and protein synthesis. It is a key player in intracellular immunity against viral infections [100,101]. The *IFI44L* gene is an interferon-induced protein with broad antiviral effects through multiple mechanisms including direct viral inhibition, modulation of immune responses, and altering cell proliferation and survival. It is an important effector molecule of interferon action against viruses. This protein also promotes macrophage differentiation and facilitates secretion of inflammatory cytokines during bacterial infection [102,103]. *RSAD2* is an interferon-induced antiviral protein with broad activity against many viruses. This protein has a role in cellular antiviral response and innate immune signaling. By regulating immune pathways, this gene stimulates antiviral cytokine production and restricts viral infection and spread [104,105]. These genes were involved in three groups (BP, MF, and CC), including regulation of biological process, regulation of cellular process, regulation of response to stimulus, response to stress, immune system process, defense response, regulation of apoptotic process, defense response to virus, binding, cellular anatomical entity, and membrane. Moreover, these were largely enriched in pathways of influenza A, NOD-like receptor, TNF, and NF-kappa B signaling pathways.

In subnets 5 and 7, *LRRC71* is an uncharacterized leucine-rich repeat-containing protein induced by type I interferons. It likely contributes to innate antiviral immunity through regulation of interferon signaling pathways. *LRRC71* may also modulate cell proliferation and apoptosis to inhibit viral infection [106,107]. The *PEG10* gene has a critical role in embryo development and placental formation by promoting cell proliferation and survival. Also, it stimulates growth pathways like Wnt/beta-catenin and inhibits TGF-beta signaling. Overexpression of this gene contributes to cancer by stimulating tumor progression and blocking apoptosis [108,109].

In subnet 6, the *NLRP3* gene encodes one of the constituent subunits of the *NLRP3* protein complex. This complex acts as an activator of NF-kappaB signaling and it is involved in regulating inflammation, immune system responses, and apoptosis [110,111]. The *CASP4* gene, also known as caspase-4, is a protein with an important role in inflammatory immune responses. When activated, *CASP4* cleaves and activates other inflammatory caspases, leading to maturation of pro-inflammatory cytokines. Through this pathway, the *CASP4* gene helps defend against bacterial pathogens by inducing cell death and inflammation [112,113]. These genes were involved in three groups (BP, MF, and CC), with roles including regulation of cellular process, regulation of response to stimulus, response to stress, immune system process, innate immune response, regulation of defense response, inflammatory response, cellular anatomical entity, extracellular region, and inflammasome complex. Moreover, pathways mainly affected by these genes include Salmonella infection and the NOD-like receptor signaling pathway.

In subnet 8, the *GPX3* gene is an antioxidant enzyme with a key role in reducing oxidative stress. This enzyme is secreted from cells into blood plasma, where it protects circulating lipids, lipoproteins, and cells from oxidative damage. It also reduces free hydrogen peroxide, preventing lipid peroxidation [114]. This gene was enriched in terms of response to stress, cellular response to chemical stimulus, protein binding, identical protein binding, cellular anatomical entity, and extracellular space.

We applied a computational approach with construction of a lncRNA–miRNA–mRNA ceRNA regulatory network using identified expression profiles (RNA-Seq and microarray) of regulatory RNAs. Spatiotemporal differential expression in blood tissue (PBMCs) supports the potential role of RNAs; furthermore, it can have a significant role in identifying candidate regulatory RNAs in transcriptional regulation involved in JD in dairy cattle. However, more studies are needed to elucidate specific molecular mechanisms and biological roles of RNA regulatory subnets related to MAP pathogenesis. Typical explanations for inconsistencies and limitations in our outputs compared to other studies were differences in molecular techniques and technologies, differences in blood tissue (PBMCs), time and environmental conditions of sampling from various laboratories and dairy cattle farms, and bioinformatics websites and tools. Regardless, integration of various regulatory RNAs based on ceRNA regulatory networks, plus lncRNA, miRNA, and mRNA interactions, provided novel insights into molecular biological processes associated with JD in dairy cattle.

## 4. Materials and Methods

**Figure 5:**
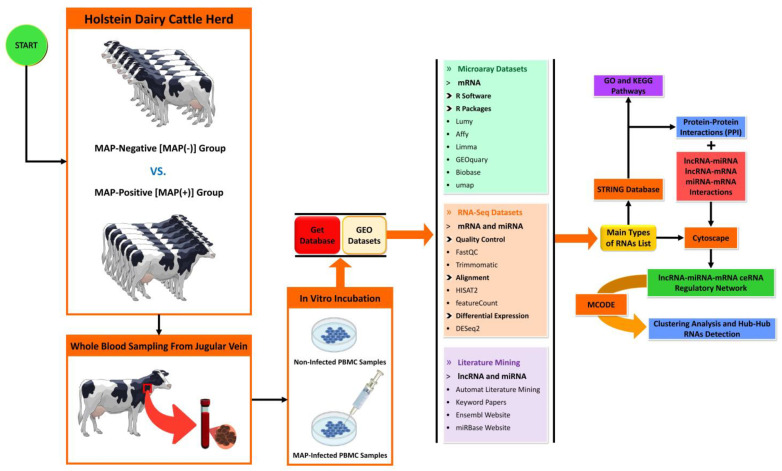
Schematic of the workflow used to construct the lncRNA–miRNA–mRNA ceRNA regulatory network involved in MAP infection. Regulatory RNAs were obtained from microarray and RNA-Seq data analyses, plus literature mining. Protein–protein interaction network (PPI), gene regulatory network (GRN), and ceRNA regulatory network were constructed using the STRING biological database and Cytoscape software.

A summary of the workflow for selecting cattle, sampling, data mining, preparation, and analysis of DE genes associated with MAP infection is presented in Figure 5.

### 4.1. Data Collection

In this study, RNA-Seq and microarray datasets were retrieved from the Gene Expression Omnibus (GEO) database from the National Center for Biotechnology Information (NCBI) and analyzed to identify candidate RNAs, determine protein-protein interactions, gene regulatory network, reconstruct a lncRNA-miRNA-mRNA ceRNA regulatory network and its subnets, plus identify relevant metabolic and signaling pathways associated with MAP infection in dairy cattle. Six GSE datasets were used. The GEO accession numbers for each RNA-Seq and microarray dataset are listed in Table 2.

The first two datasets, GSE62048 and GSE35185, included seven age-matched Holstein-Friesian cows, 4 years old, from a herd managed by the UCD Lyons Research Farm (Newcastle, County Kildare, Ireland) without a history of JD; these datasets were related to microarray analysis from a study by MacHugh et al. [20] and an RNA-Seq analysis performed by Casey et al. [21], respectively. All cows were similarly housed and fed the same diets at the UCD Lyon Research Farm (Newcastle, County Kildare, Ireland). Briefly, monocyte-derived macrophages (MDM) were purified from PBMCs prepared from whole blood extracted from seven Holstein-Friesian cows. Samples were divided into MAP-infected and MAP-negative control groups. Total RNA was extracted and purified from each sample at 0, 2, and 6 h post-infection (hpi) and used to prepare microarray and RNA-seq libraries [20,21].

In the third dataset, GSE122933, all cattle tested negative for MAP infection by serum ELISA and fecal culture (Laboklin, Bad Kissingen, Germany). Then, whole-blood samples from three 15-month-old Holstein-Friesian heifers were collected. Samples were collected from the jugular vein under sterile conditions and stored in Na-citrate to prevent coagulation. Then, PBMCs were isolated by centrifugation, washed with PBS, and allowed to attach to the cell-culture plate surface. Six samples underwent total RNA sequencing at the Institute of Clinical Molecular Biology at Christian-Albrechts-University Kiel, where both MAP-infected and control samples consisted of three biological replicates [6].

In the fourth dataset, GSE149494, all cattle were continuously MAP ELISA-negative (IDEXX Laboratories, Inc., Westbrook, ME, USA). The selected cows were housed on an experimental farm of Seoul National University. Bovine PBMCs were isolated from a Holstein cow that had tested negative by commercial ELISA kit for JD. Madin–Darby bovine kidney (MDBK) epithelial cells were infected with MAP and incubated for 4 h to allow MAP traversal across the epithelial monolayer. After incubation, MAP bacteria were isolated from the basal chamber using differential centrifugation and purification steps. Subsequently, bovine PBMCs were infected with native MAP (group T1) and MDBK-processed MAP (group T2) for 24, 72, and 120 h [4].

The fifth dataset, GSE98363, contained data from 12 cows from commercial dairy herds in the province of Québec, Canada. All cows were tested for MAP infection by serum ELISA and fecal culture tests. Six cows with no previous history of JD that tested negative on both the serum ELISA and fecal culture were considered MAP-negative (MAP(−)), and another six cows, all from MAP(+) herds, that tested positive on both tests, were considered MAP-positive (MAP(+)). Blood samples were obtained from the jugular vein and PBMCs were isolated from 700 mL of blood and purified. The experimental design of the MDM infecting with MAP used incubation periods of 1, 4, 8, and 24 h [115].

The sixth dataset, GSE62835, was related to microarray analysis from Shin et al. [116]. Eight cows were used for differential gene expression analysis between MAP-infected and MAP-negative cows. Cows were allocated into four groups: 1) control group (*n* = 2) MAP ELISA and fecal PCR negative; 2) test1 group (*n* = 2) MAP ELISA negative, but PCR positive; 3) test2 group (*n* = 2), MAP ELISA-positive but PCR negative; and 4) test3 group (*n* = 2) MAP ELISA and fecal PCR-positive. Peripheral blood samples were collected from each cow and PBMCs isolation and purification were performed.

### 4.2. Differential Gene Expression Analysis

Initial processing and normalization of microarray data were conducted using the Lumi package [117] and the GCRMA (GeneChip Robust Multi-Array Averaging) algorithm method implemented in the Affy package in R software (v. 4.3) in order to prepare data for further analyses [118]. Gene expression analysis was performed to identify significant differentially expressed (DE) mRNAs/genes in R/bioconductor software (v. 3.19) between MAP-positive and MAP-negative sample groups using Limma [119], GEOquary [120], Biobase [121], and Umap [122] packages.

For RNA-Seq data, the quality of raw fastq data was checked using FastQC software (v0.11.9) [123]. Based on quality control results, adapters, PCR primers, and low-quality reads were removed from the sequences using Trimmomatic (v 0.38.0) [124]. Cleaned reads were aligned on the *Bos taurus* reference genome (http://ftp.ensembl.org/pub/release-103/fasta/bos_taurus/dna/ accessed on 12 December 2023) with HISAT2 (v 2.2.1) [125]. FeatureCounts (v 2.0.3) software was used to quantify transcripts by measuring total read counts of mapped sequences [126]. Differential gene expression analysis was conducted in DESeq2 (v2.11.40.7) [127]. Concurrent identification of miRNAs was also performed on RNA-Seq data. Differentially expressed mRNAs/genes were determined using thresholds of |log2 fold change| ≥ 1 and false discovery rate (FDR) ≤ 0.05. Microarray and RNA-Seq differential expression gene lists constituted Gene Lists 1 and 2, respectively (Supplementary Table S2).

### 4.3. Literature Mining to Discover Candidate miRNAs and lncRNAs Related to Johne’s Disease

Various online databases were searched to identify candidate miRNAs and lncRNAs relevant to JD in *Bos taurus*. The literature search was conducted using Google Scholar, PubMed, Web of Science, and CrossRef from 2010 to 2023 without language restrictions. Search terms included both keywords and database-specific subject headings related to ceRNA regulatory networks and bovine paratuberculosis or JD: breeds—dairy cattle; technology—microarray and RNA-Seq; outcomes—ceRNA network or regulatory RNAs; and trait—immune response to MAP. Additional keywords were *Mycobacterium avium* subsp. *paratuberculosis* (MAP), Monocyte-Derived Macrophages, Johne’s disease, lncRNA, miRNA, and ceRNA regulatory networks. First, identifiers and synonyms for each search element were combined using the Boolean operator “OR”. Second, search elements were merged using the Boolean operator “AND”. Identified non-coding RNAs (miRNAs and lncRNAs) are listed in Supplementary Table S3.

While reviewing the literature, we created a significant and key gene list that interacts with genes identified in this study and complements identification of important metabolic-signaling pathways relevant to MAP infection in dairy cattle. In contrast to previously published studies, we used literature mining to identify and collect all types of non-coding RNAs and their interactions with identified mRNAs and miRNAs in our study. This led to reconstruction of the ceRNA regulatory network, improving understanding of the gene regulation mechanisms related to JD in dairy cattle.

### 4.4. Determining the Main RNAs List

To identify candidate mRNAs and miRNAs related to MAP infection, the number of common DE RNAs from RNA-Seq and microarray GSE accessions were analyzed using the “Calculate and draw custom Venn diagrams” online tool (https://bioinformatics.psb.ugent.be/webtools/Venn/ accessed on 21 December 2023), separately, and then merged, as seen in Supplementary Table S4. Subsequently, the list of ncRNAs (Supplementary Table S3) from literature mining was integrated with Supplementary Table S4 (from RNA-Seq and microarray analysis) as the main RNAs list (Supplementary Table S5).

### 4.5. Functional Enrichment Analysis and KEGG Pathways

Gene ontology (GO) and enrichment analysis were conducted using online tools DAVID (Database for Annotation, Visualization, and Integrated Discovery; [128]), PANTHER (Protein Analysis Through Evolutionary Relationships; [129]), GeneCards (www.genecards.org/ accessed on 23 December 2023), and STRING database ([130]; https://string-db.org accessed on 23 December 2023), comprehensive online web tools to explore the biological process (BP), molecular function (MF), and cellular component (CC) related to the main RNAs list. Pathway enrichment analysis of identified mRNAs/genes was conducted using the Kyoto Encyclopedia of Genes and Genomes (KEGG). GO terms with a false discovery rate (FDR) < 0.05 were considered significantly enriched for the identified mRNAs/genes.

### 4.6. Identification of Regulatory RNAs and Target Gene Prediction

Potential miRNA target predictions were performed using the miRBase ([131]; https://www.mirbase.org/ accessed on 24 December 2023) and miRWalk (http://mirwalk.umm.uni-heidelberg.de/ accessed on 24 December 2023) databases. Targeted mRNAs were submitted to the DAVID and STRING databases to identify enriched targets for each miRNA. Other targeted interactions between regulatory RNAs were predicted using the LNCipedia ([132]; https://lncipedia.org accessed on 24 December 2023) and NONCODE ([133]; http://www.noncode.org/ accessed 24 December 2023) databases.

### 4.7. Reconstruction of lncRNA–miRNA–mRNA ceRNA Regulatory Network and Clustering Analysis

The lncRNA-miRNA-mRNA ceRNA regulatory network was reconstructed based on documented lncRNA-mRNA, lncRNA-miRNA, and miRNA-mRNA interactions from related articles and online interaction databases. Protein-protein interaction (PPI) data were gathered and gene regulatory network (GRN) analyses were performed using the STRING database. STRING integrates interaction data from seven primary sources, including neighborhood, co-occurrence, fusion, experimental, co-expression, databases, and text mining, to generate confidence scores for protein-protein interactions [130]. Cytoscape (v 3.8.2) is an offline tool with plugins for data integration, screening, and visualization [134]. In the ceRNA regulatory network, biological molecules (RNAs) and their interactions were represented as nodes and edges, respectively. MCODE is a Cytoscape plugin used to identify functional clusters and hub nodes in the lncRNA–miRNA–mRNA ceRNA regulatory network. The MCODE plugin can detect clusters in directed or undirected networks [43]. Regions of the network with highly interconnected nodes were considered clusters. The gene expression network was constructed at β = 12 to ensure scale-free topology (R2 ≥ 0.8). If the scale-free topology fit index exceeds 0.8 at powers <30 for a reference dataset, the network topology is considered scale-free and devoid of batch effects [135,136,137,138]. Furthermore, enrichment of metabolic and signaling pathways in the ceRNA regulatory network and subnetworks was identified using STRING, DAVID, and PANTHER.

## 5. Conclusions

The present study used a method that is novel in animal genetics to combine various regulatory RNAs as a multi-partite network related to JD in dairy cattle. Integrating transcriptome profiles (RNA-Seq and microarray) from differential expression analysis of MAP-positive and MAP-negative sample groups to provide hub RNAs with differential expression levels identified 28 lncRNAs, 42 miRNAs, and 370 mRNA/genes involved in MAP infection. In this regard, we identified 21 hub genes involved in MAP infection. According to the lncRNA–miRNA–mRNA ceRNA regulatory network, eight significant subnets with a total of eight lncRNAs, 29 miRNAs, and 237 mRNAs/genes were identified as being involved in major biological molecular mechanisms, including immune system process, defense response, response to cytokine, leukocyte migration, regulation of T cell activation, defense response to bacterium, NOD-like receptor, B cell receptor, TNF, NF-kappa B, IL-17, and T cell receptor signaling pathways. Spatio-temporal differential expression in various tissues, especially in PMBCs, supported the potential role of coding and non-coding RNAs in transcriptional and post-transcriptional regulation of genes and may be beneficial in discovering basic molecular regulatory mechanisms of bovine MAP infection. Consequently, our results demonstrated that integrated transcriptome profiles and ceRNA regulatory network analyses between various MAP-positive and MAP-negative samples for PMBCs for identification of hub genes, pathways, and their respective functions could generate novel insights to improve understanding of the genetics and mechanisms regulating MAP infection and can be considered a starting point for future studies on bovine MAP infection. Regardless, more research is needed to validate these hub RNAs in this disease.

## Figures and Tables

**Table 1:** Differentially expressed miRNAs between samples from MAP-positive and MAP-negative dairy cattle.

miRNA Name	miRNA Locus			Fold Changes (FC)	*p*-Value	FDR
	BTA	miRNA Start	miRNA End			
bta-mir-125b-1	15	32763901	32763988	4.6941	0.0047	0.0219
bta-mir-140	18	36962003	36962116	−1.0828	0.0107	0.0391
bta-mir-17	12	65689417	65689500	1.0932	0.0024	0.0139
bta-mir-2285g-2	10	30425778	30425855	2.17155	0.0028	0.0152
bta-mir-2285l	2	103419041	103419122	4.1647	0.0034	0.0176
bta-mir-2285o-5	5	117234119	117234194	2.3742	0.0041	0.0199
bta-mir-2315	15	42178557	42178636	1.2995	0.0003	0.0031
bta-mir-2351	2	9689788	9689863	1.2942	0.0014	0.0094
bta-mir-2384	25	18625328	18625400	1.19179	0.0001	0.0016
bta-mir-2398	27	19351413	19351491	−2.5526	0.0079	0.0314
bta-mir-301a	19	10121293	10121377	2.4534	0.0056	0.0247
bta-mir-302a	6	12980768	12980837	1.4491	0.0068	0.0283
bta-mir-302c	6	12980357	12980424	1.5567	0.0037	0.0187
bta-mir-339a	25	41736134	41736211	−1.5179	0.0062	0.0267
bta-mir-4657	4	76669524	76669579	1.0202	0.0006	0.0051
MIRLET7F1	8	85450736	85450845	1.1559	0.0008	0.0063
bta-mir-365-2	19	18461502	18461612	3.5664	0.0035	0.0207
bta-mir-147	10	65015857	65015936	1.1365	0.0021	0.0124
bta-mir-371	18	60903171	60903249	1.9198	0.0121	0.0423

**Table 2:** Summary of GEO accession numbers for RNA-Seq and microarray data sets associated with JD.

No.	Data Type	GEO ^a^ Accession	Platform	Samples (MAP:H) ^b^	References
1	RNA-Seq	GSE62048	GPL15749 (Illumina HiSeq 2000 (*Bos taurus*))	35 (14:21)	[21]
2	Microarray	GSE35185	GPL2112((Bovine) Affymetrix Bovine Genome Array)	48 (20:28)	[20]
3	RNA-Seq	GSE122933	GPL23295 (Illumina HiSeq 4000 (*Bos taurus*))	6 (3:3)	[6]
4	RNA-Seq	GSE149494	GPL26012 (Illumina NovaSeq 6000 (Bos taurus))	12 (8:4)	[4]
5	RNA-Seq	GSE98363	GPL15749 (Illumina HiSeq 2000 (*Bos taurus*))	24 (12:12)	[2,115]
6	Microarray	GSE62835	GPL11649 (Agilent-023647 *B. taurus* (Bovine) Oligo Microarray v2 (Probe Name version))	6 (9:3)	[116]

^a^ GEO, Gene Expression Omnibus; ^b^ MAP, number of *Mycobacterium avium* subsp. *paratuberculosis* samples, and H, number of MAP-negative samples.

## Data Availability

Datasets used in this study are publicly available and can be accessed from the National Center for Biotechnology Information (NCBI) with BioProject numbers PRJNA150677, PRJNA265814, PRJNA506960, PRJNA628877, PRJNA384849 and PRJNA263027. Further details on accessing the data are on the NCBI website at https://www.ncbi.nlm.nih.gov/bioproject/ accessed on 10 November 2023.

## Supplementary Materials

The following supporting information can be downloaded at: https://www.mdpi.com/article/10.3390/ncrna10040038/s1., Table S1: Basic parameters of protein-protein interaction (PPI) network on Johne’s disease (JD) in dairy cattle; Table S2: Significantly differentially expressed genes in peripheral blood mononuclear cells (PBMCs) between MAP-positive and MAP-negative sample groups for Johne’s disease (JD); Table S3: Candidate non-coding RNAs (i.e., lncRNAs, and miRNAs) list extracted from transcriptome results using literature mining on Johne’s disease (JD) in dairy cattle; Table S4: Common differentially expressed RNAs between MAP-positive and MAP-negative sample groups for Johne’s disease (JD) from RNA-Seq analysis and microarray data sets; Table S5: Main RNAs extracted from transcriptome profiles (RNA-Seq and microarray) analysis and literature mining on Johne’s disease (JD) in dairy cattle; Table S6: Basic parameters of biological process (BP), molecular function (MF), and cellular component (CC) pathways on Johne’s disease (JD) in dairy cattle; Table S7: Basic parameters of top KEGG pathways on Johne’s disease (JD) in dairy cattle; Figure S1: The ceRNA regulatory network included 28 lncRNAs, 42 DE miRNAs and 370 DE mRNAs. Octagonal nodes represent lncRNAs, triangle nodes represent miRNAs, and quadrilateral nodes represent mRNAs/genes. Black edges indicate interactions between nodes; Figure S2: Subnet 1: 2 lncRNAs, 16 DE miRNAs and 170 DE mRNAs in an interacted network were identified. In this subnet, octagonal nodes represent lncRNAs, triangle nodes represent miRNAs, and quadrilateral nodes represent mRNAs/genes. The orange quadri-lateral represents hub mRNAs/genes involved in the subnet. Edges indicate interactions; black edges represent mRNA–mRNA interactions, green edges represent lncRNA–mRNA interactions, and red edges represent miRNA–mRNA interactions; Figure S3: Subnet 2: 6 lncRNAs, 18 DE miRNAs and 101 DE mRNAs in an interacted network were identified. In this subnet, octagonal nodes represent lncRNAs, triangle nodes represent miRNAs, and quadrilateral nodes represent mRNAs/genes. The orange quadri-lateral represents hub mRNAs/genes involved in the subnet. Edges indicate interactions; black edges represent mRNA–mRNA interactions, green edges represent lncRNA–mRNA interactions, and red edges represent miRNA–mRNA interactions; Figure S4: Subnet 3: 3 lncRNAs, 9 DE miRNAs, and 82 DE mRNAs in an interacted network were identified. In this subnet, octagonal nodes represent lncRNAs, triangle nodes represent miRNAs, and quadrilateral nodes represent mRNAs/genes. The orange quadri-lateral represents hub mRNAs/genes involved in the subnet. Edges indicate interactions; black edges represent mRNA–mRNA interactions, green edges represent lncRNA–mRNA interactions, and red edges represent miRNA–mRNA interactions; Figure S5: Subnet 4: three lncRNAs, nine DE miRNAs and 35 DE mRNAs in an interacted network were identified. In this subnet, octagonal nodes represent lncRNAs, triangle nodes represent miRNAs, and quadrilateral nodes represent mRNAs/genes. The orange quadrilateral represents hub mRNAs/genes involved in the subnet. Edges indicate inter-actions; black edges represent mRNA–mRNA interactions, green edges represent lncRNA–mRNA interactions, and red edges represent miRNA–mRNA interactions; Figure S6: Subnet 5: 10 DE miRNAs and 35 DE mRNAs in an interacted network were identified. In this subnet, triangle nodes represent miRNAs, and quadrilateral nodes represent mRNAs/genes. The orange quadrilateral represents hub mRNAs/genes involved in the subnet. Edges indicate interactions; black edges represent mRNA–mRNA interactions and red edges represent miRNA–mRNA interactions; Figure S7: Subnet 6: five DE miRNAs and 23 DE mRNAs in an interacted network were identified. In this subnet, triangle nodes represent miRNAs, and quadrilateral nodes represent mRNAs/genes. The orange quadrilateral represents hub mRNAs/genes involved in the subnet. Edges indicate interactions; black edges represent mRNA–mRNA interactions and red edges represent miRNA–mRNA interactions; Figure S8: Subnet 7: six DE miRNAs and 20 DE mRNAs in an interacted network were identified. In this subnet, triangle nodes represent miRNAs, and quadrilateral nodes represent mRNAs/genes. The orange quadrilateral represents hub mRNAs/genes involved in the subnet. Edges indicate interactions; black edges represent mRNA–mRNA interactions and red edges represent miRNA–mRNA interactions; Figure S9: Subnet 8: six DE miRNAs and 17 DE mRNAs in an interacted network were identified. In this subnet, triangle nodes represent miRNAs, and quadrilateral nodes represent mRNAs/genes. The orange quadrilateral represents hub mRNAs/genes involved in the subnet. Edges indicate interactions; black edges represent mRNA–mRNA interactions and red edges represent miRNA–mRNA interactions; References [5,6,10,44,139] are cited in Supplementary Materials.
