# Supplementary figures and images for: Integrated Analysis of Transcriptome Profiles and lncRNA–miRNA–mRNA Competing Endogenous RNA Regulatory Network to Identify Biological Functional Effects of Genes and Pathways Associated with Johne’s Disease in Dairy Cattle
Source: Noncoding RNA. 2024 Jun 28;10(4):38. doi: 10.3390/ncrna10040038 (PMC11270299; doi:10.3390/ncrna10040038)

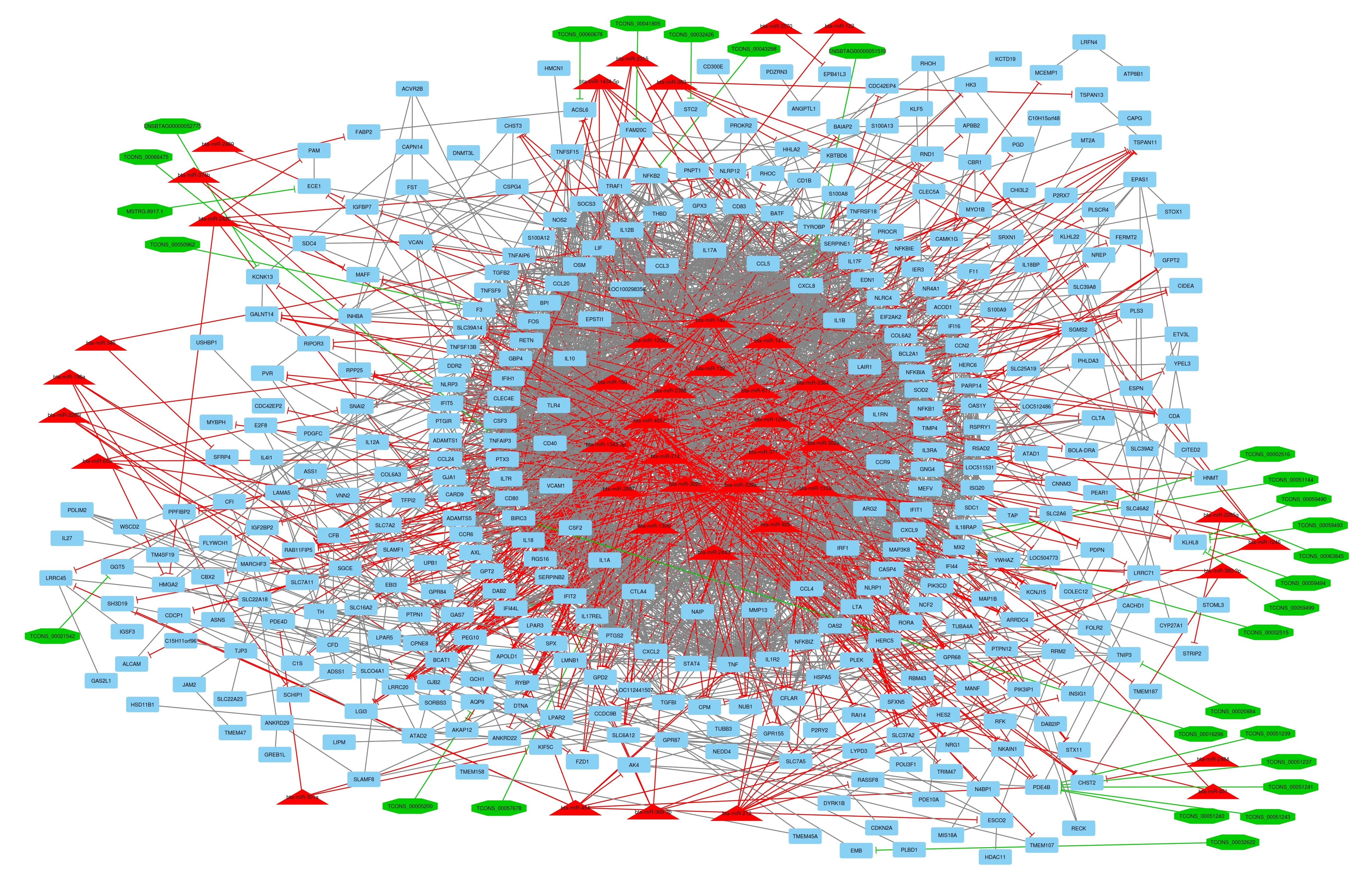

Supplement: Supplementary file 1 [file ncrna-10-00038-s001.zip › ncrna-2986275-Supplementary Materials/Figures/Supplementary Materials Figure S1.jpg]

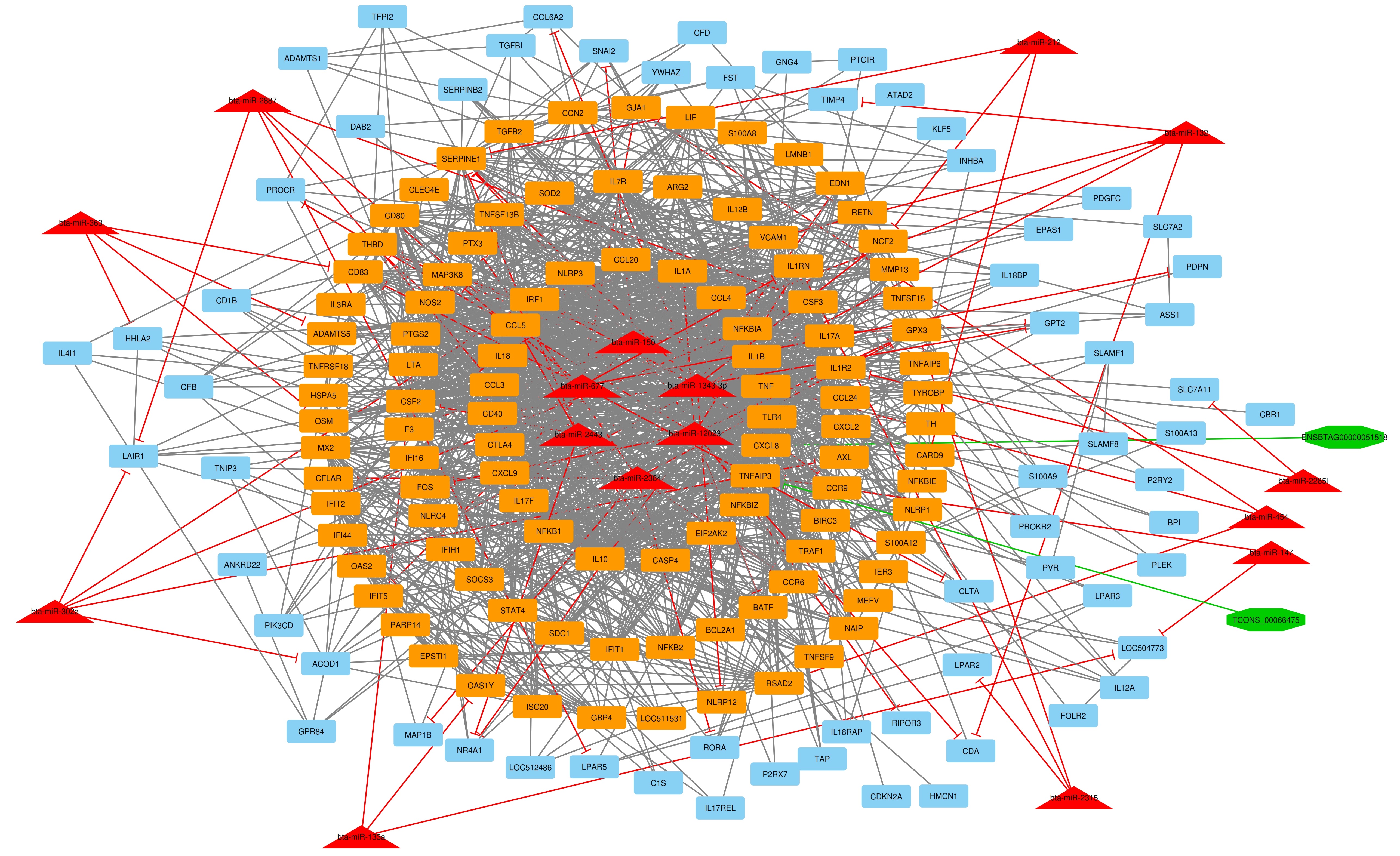

Supplement: Supplementary file 1 [file ncrna-10-00038-s001.zip › ncrna-2986275-Supplementary Materials/Figures/Supplementary Materials Figure S2.jpg]

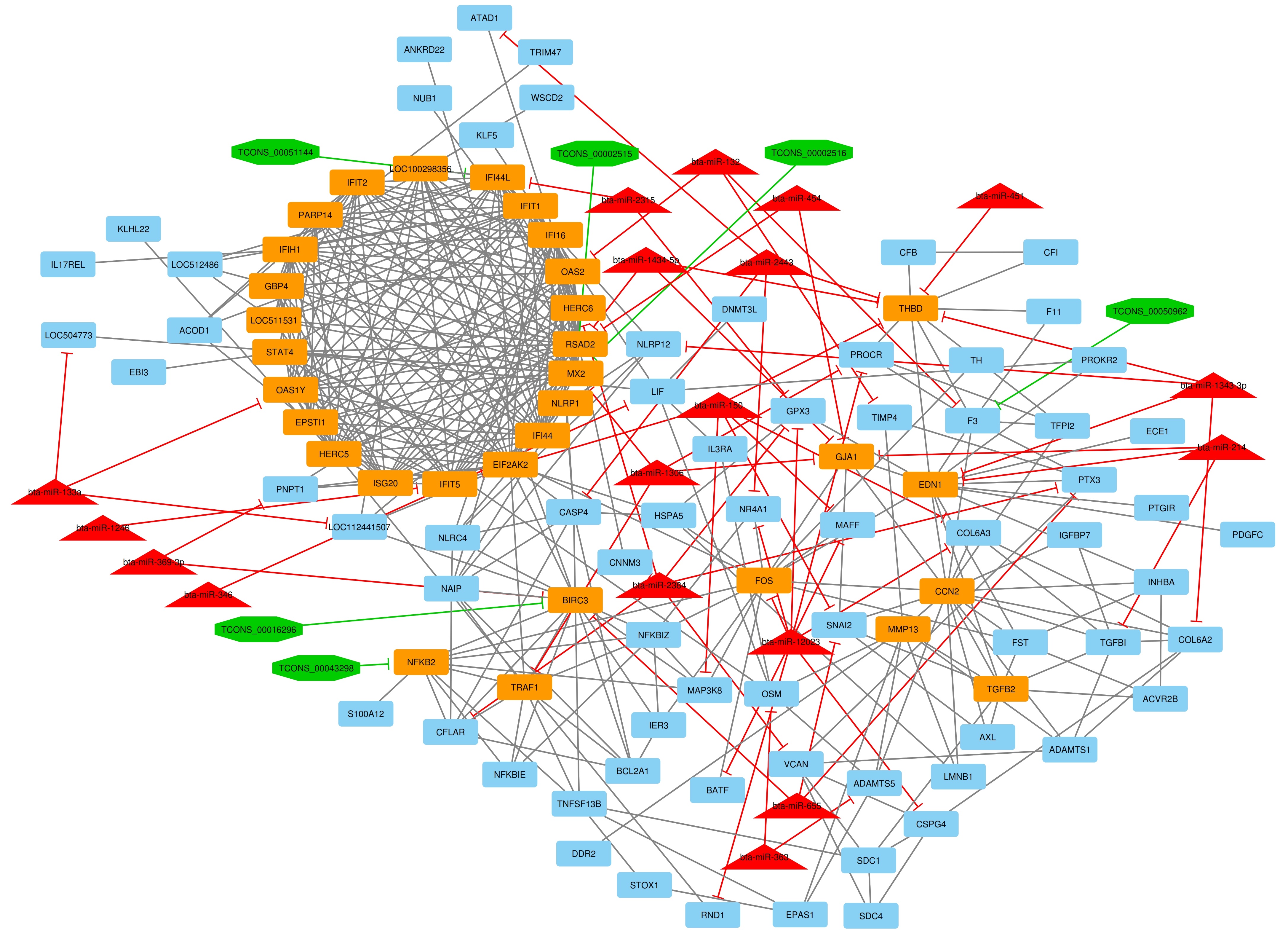

Supplement: Supplementary file 1 [file ncrna-10-00038-s001.zip › ncrna-2986275-Supplementary Materials/Figures/Supplementary Materials Figure S3.jpg]

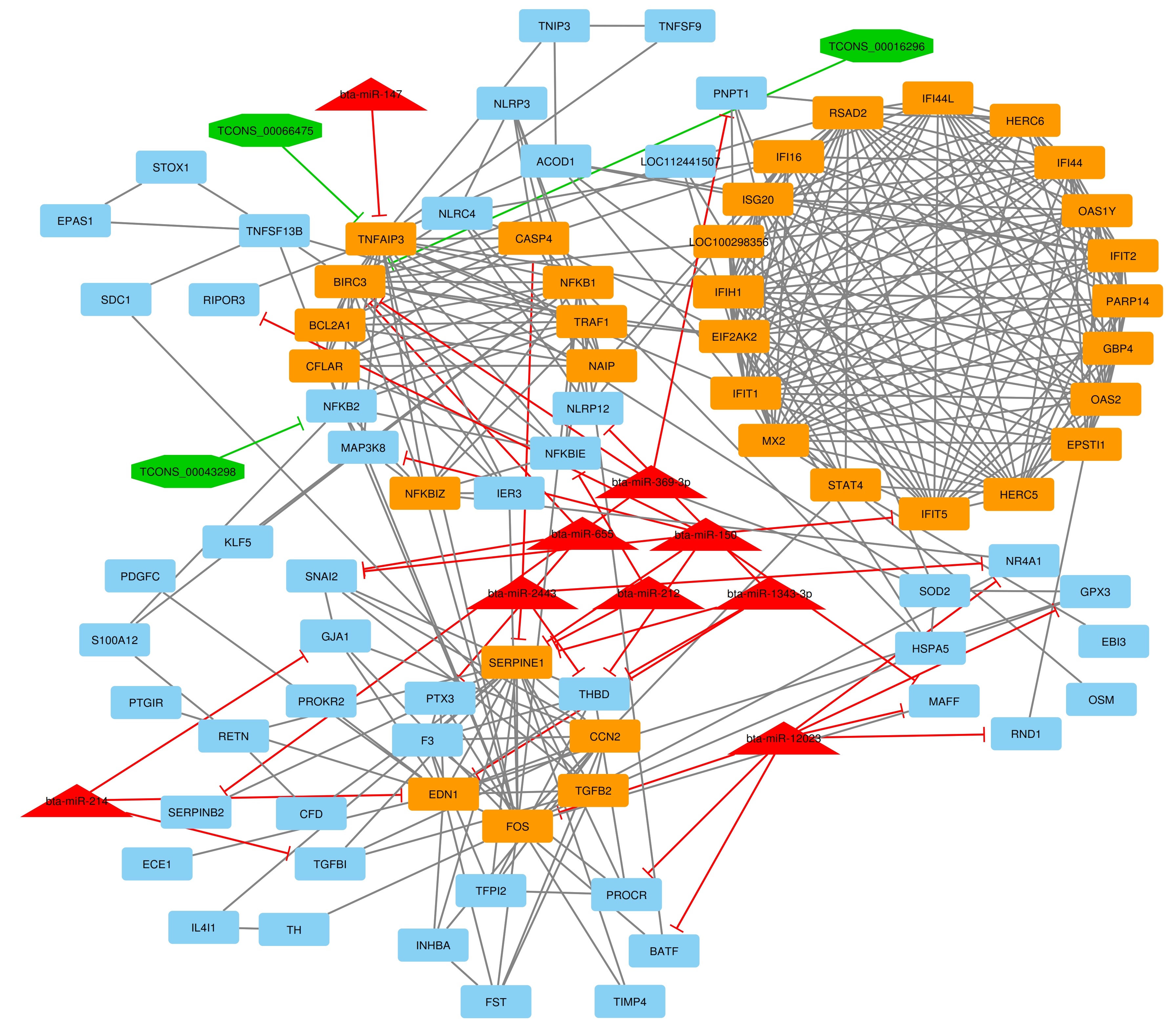

Supplement: Supplementary file 1 [file ncrna-10-00038-s001.zip › ncrna-2986275-Supplementary Materials/Figures/Supplementary Materials Figure S4.jpg]

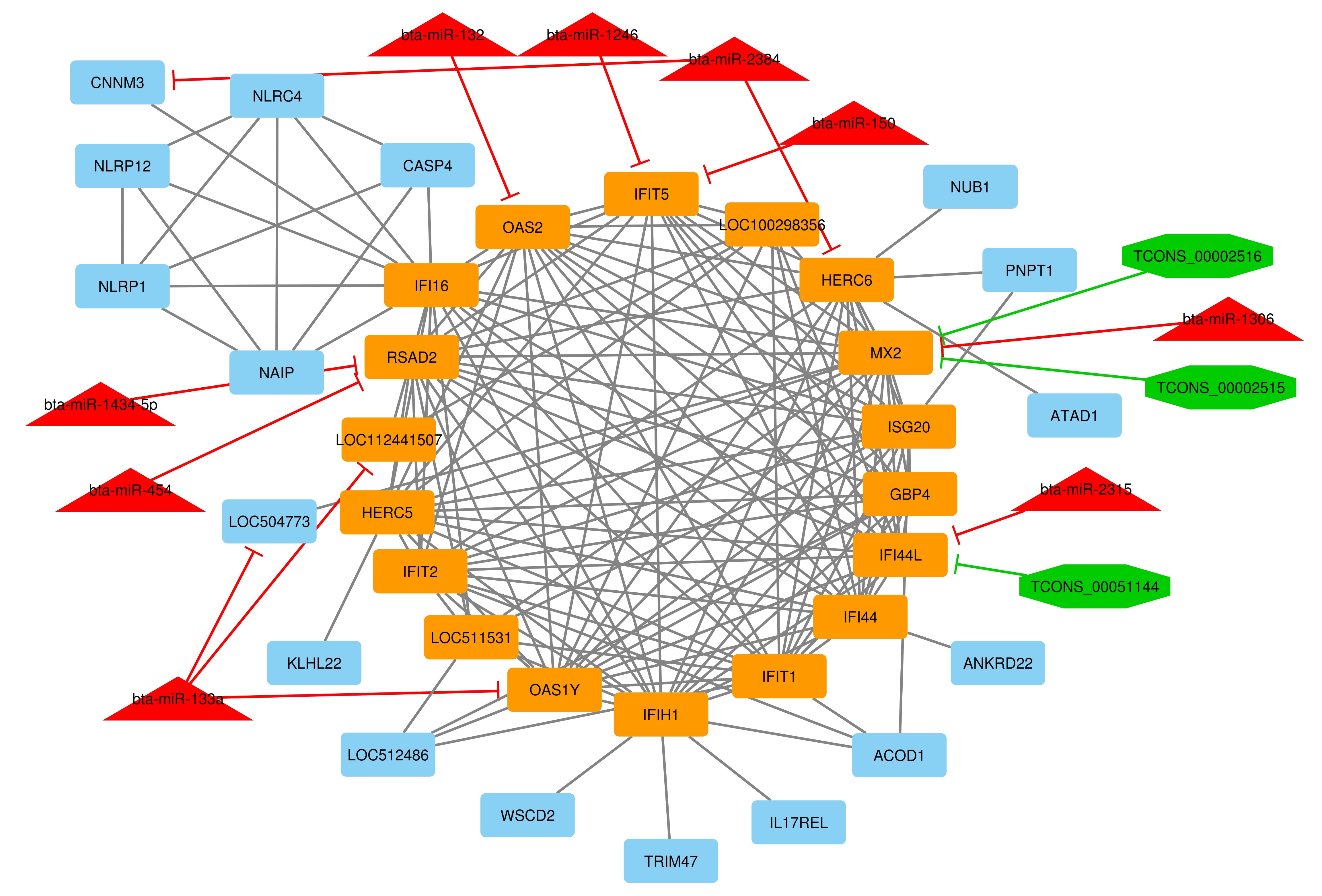

Supplement: Supplementary file 1 [file ncrna-10-00038-s001.zip › ncrna-2986275-Supplementary Materials/Figures/Supplementary Materials Figure S5.jpg]

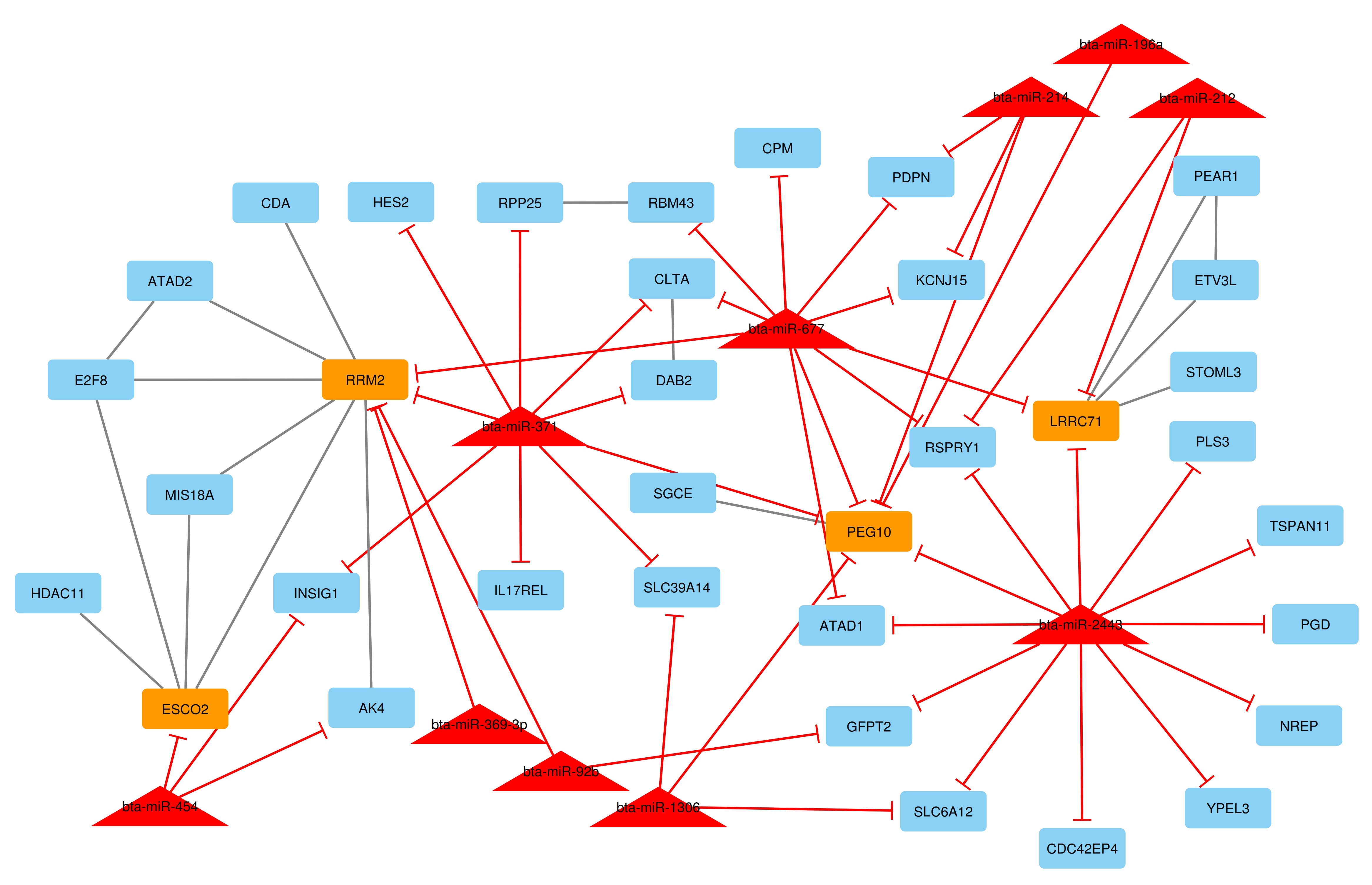

Supplement: Supplementary file 1 [file ncrna-10-00038-s001.zip › ncrna-2986275-Supplementary Materials/Figures/Supplementary Materials Figure S6.jpg]

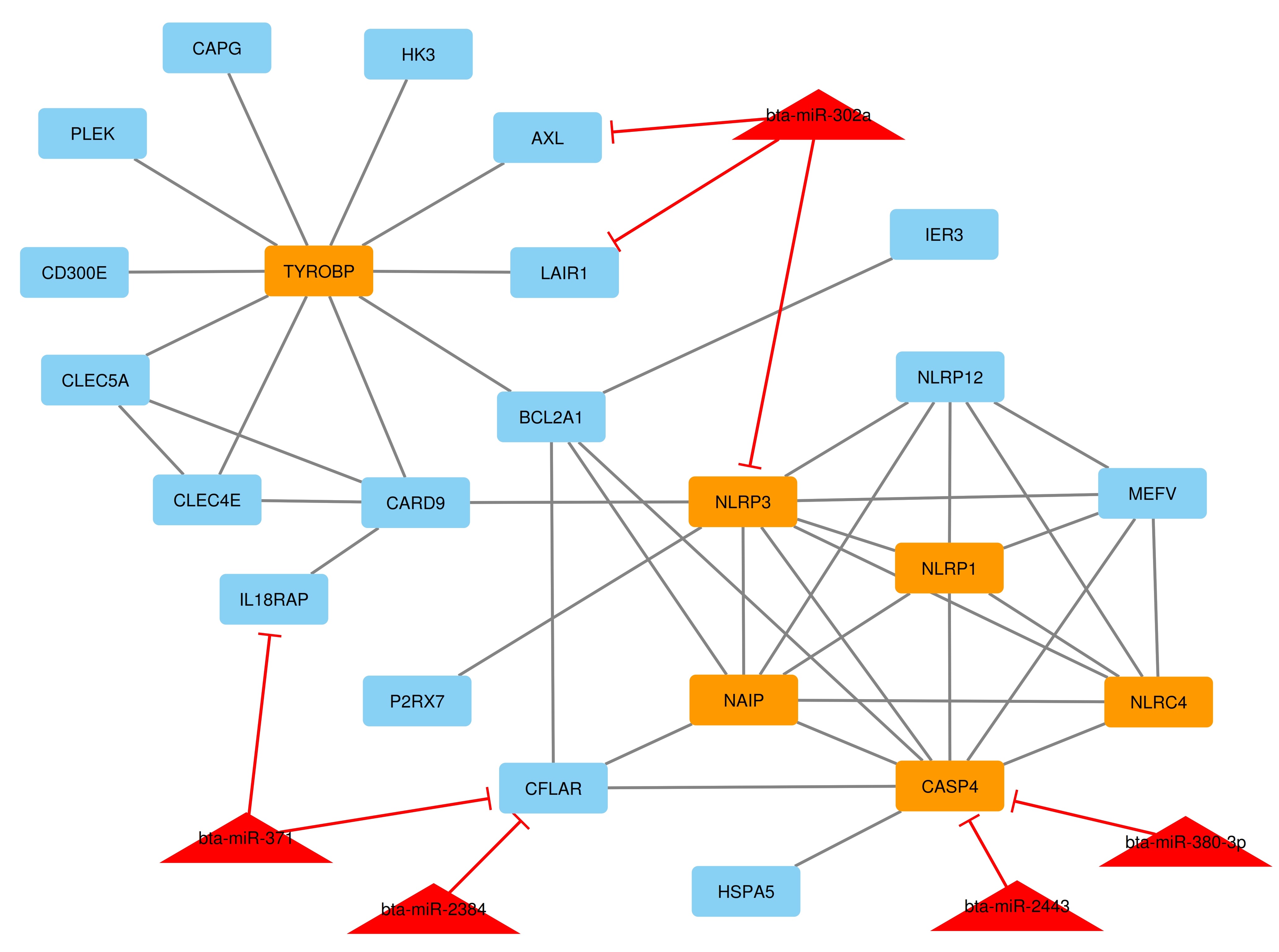

Supplement: Supplementary file 1 [file ncrna-10-00038-s001.zip › ncrna-2986275-Supplementary Materials/Figures/Supplementary Materials Figure S7.jpg]

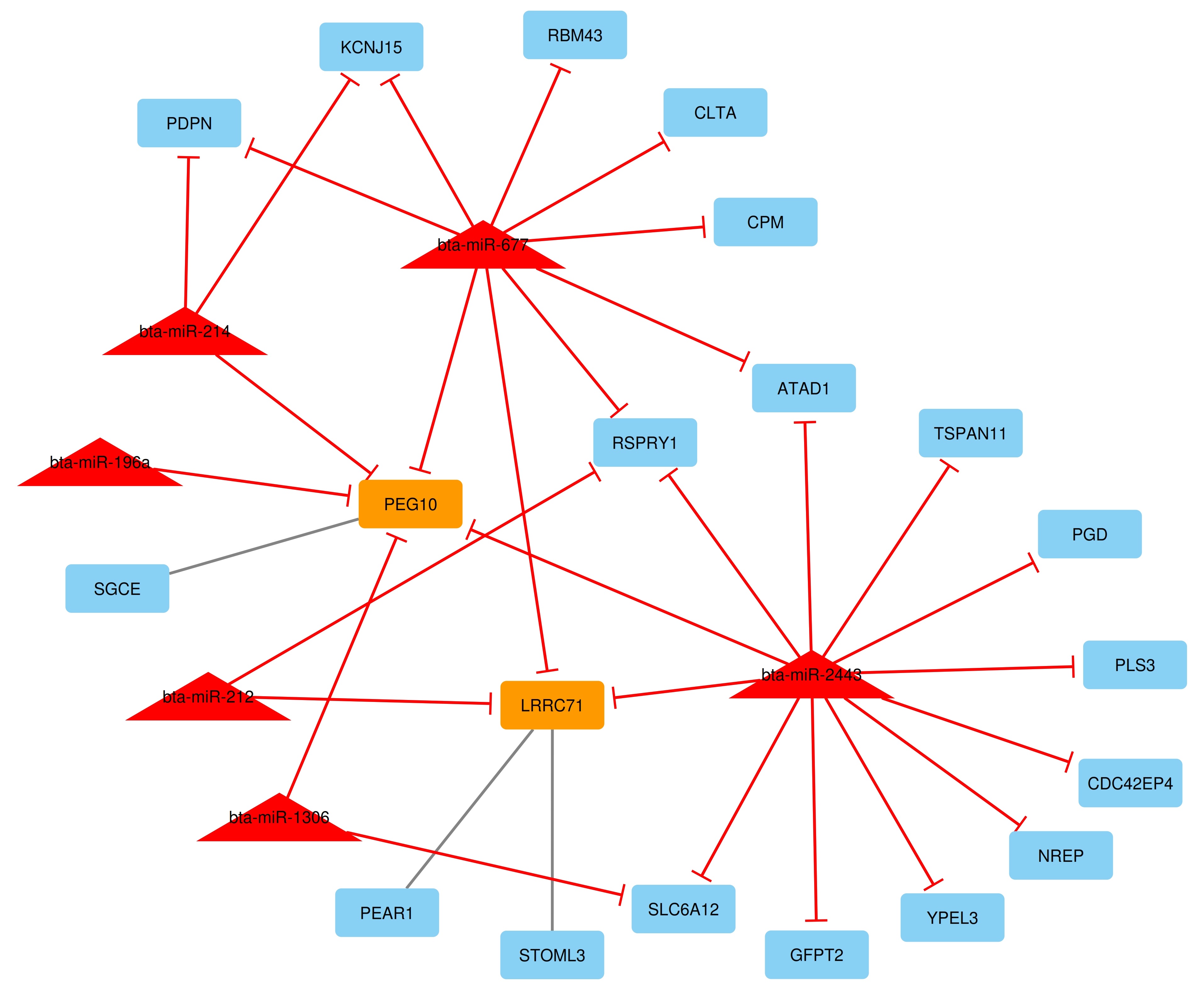

Supplement: Supplementary file 1 [file ncrna-10-00038-s001.zip › ncrna-2986275-Supplementary Materials/Figures/Supplementary Materials Figure S8.jpg]

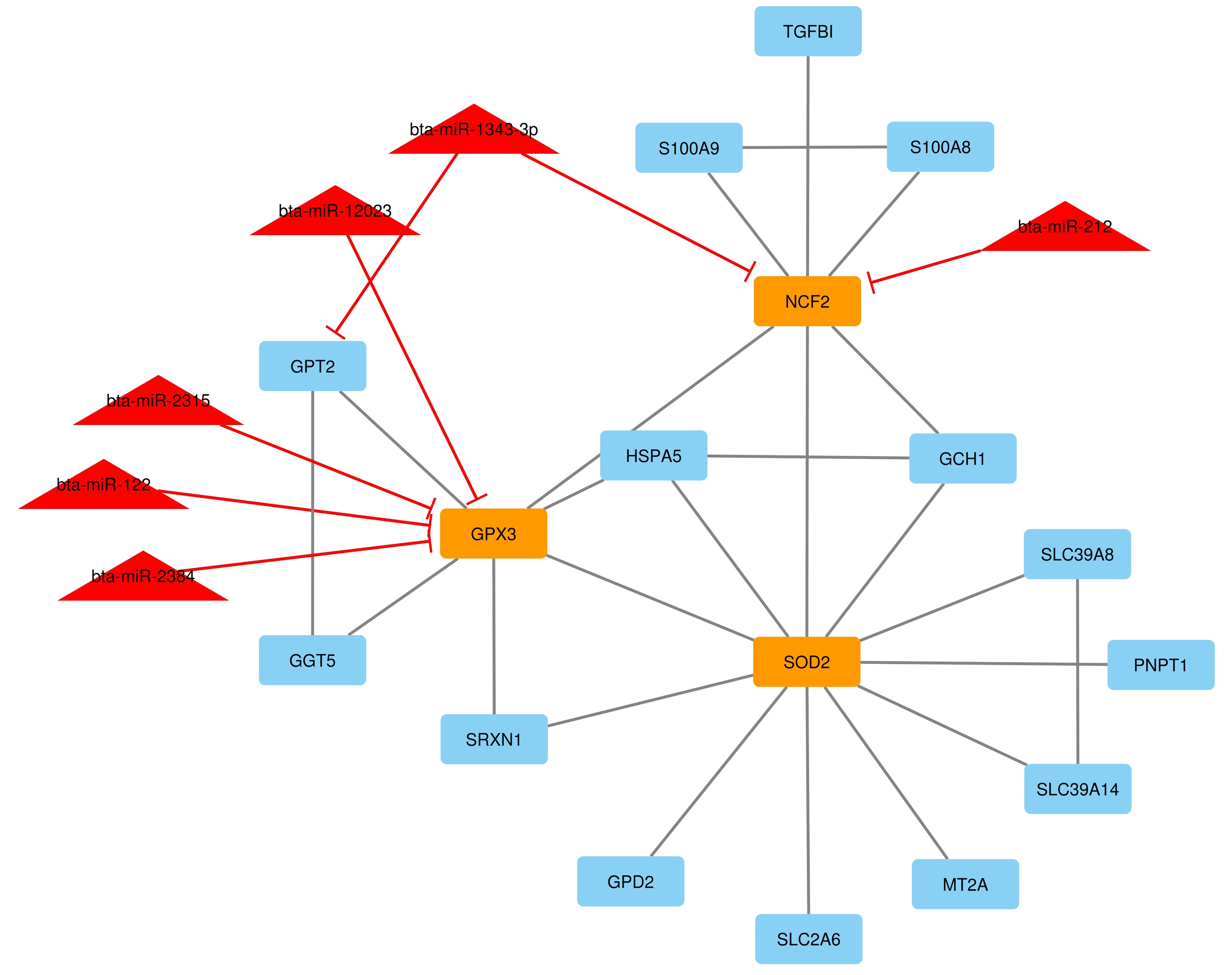

Supplement: Supplementary file 1 [file ncrna-10-00038-s001.zip › ncrna-2986275-Supplementary Materials/Figures/Supplementary Materials Figure S9.jpg]
